# Foxh1/Nodal Defines Context-Specific Direct Maternal Wnt/β-Catenin Target Gene Regulation in Early Development

**DOI:** 10.1016/j.isci.2020.101314

**Published:** 2020-06-25

**Authors:** Boni A. Afouda, Yukio Nakamura, Sophie Shaw, Rebekah M. Charney, Kitt D. Paraiso, Ira L. Blitz, Ken W.Y. Cho, Stefan Hoppler

**Affiliations:** 1 Institute of Medical Sciences, Foresterhill Health Campus, University of Aberdeen, ABERDEEN AB25 2ZD Scotland, UK; 2 Centre for Genome Enabled Biology and Medicine, Old Aberdeen Campus, University of Aberdeen, ABERDEEN AB24 2FX Scotland, UK; 3 Department of Developmental and Cell Biology, School of Biological Sciences, University of California, Irvine, CA 92697, USA

**Keywords:** Biological Sciences, Molecular Biology, Developmental Biology

## Abstract

Although Wnt/β-catenin signaling is generally conserved and well understood, the regulatory mechanisms controlling context-specific direct Wnt target gene expression in development and disease are still unclear. The onset of zygotic gene transcription in early embryogenesis represents an ideal, accessible experimental system to investigate context-specific direct Wnt target gene regulation. We combine transcriptomics using RNA-seq with genome-wide β-catenin association using ChIP-seq to identify stage-specific direct Wnt target genes. We propose coherent feedforward regulation involving two distinct classes of direct maternal Wnt target genes, which differ both in expression and persistence of β-catenin association. We discover that genomic β-catenin association overlaps with Foxh1-associated regulatory sequences and demonstrate that direct maternal Wnt target gene expression requires Foxh1 function and Nodal/Tgfβ signaling. Our results support a new paradigm for direct Wnt target gene co-regulation with context-specific mechanisms that will inform future studies of embryonic development and more widely stem cell-mediated homeostasis and human disease.

## Highlights

• Combining RNA-seq and β-catenin ChIP-seq identifies direct Wnt target genes
• Two distinct classes of direct maternal Wnt/β-catenin target genes can be discerned
• We propose coherent feedforward regulation of gene expression of the second class
• Maternal Wnt target gene expression of both classes requires Nodal/Foxh1 signaling

## Graphical Abstract

**Figure:**
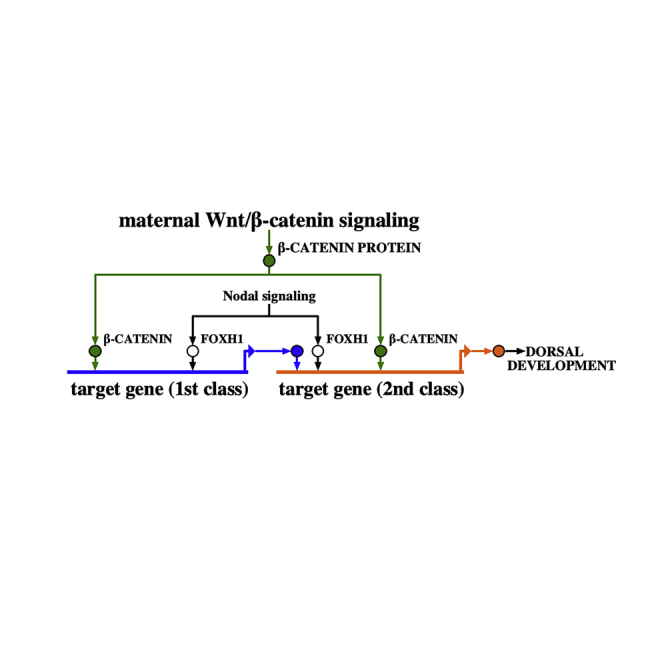

## Introduction

The maternal-to-zygotic transition activates transcription of gene batteries under the control of transcription factors and signaling pathway components that are deposited in the egg by the maternal genome. Zygotic gene activation (ZGA) is initially controlled solely by these maternal factors, but maternal control is handed over to the zygotic genome following the synthesis of new gene products. How genes are differentially regulated by transcription factors to specify tissue-specific progenitor cells during this transition is an area of active investigation (reviewed by Nakamura and Hoppler, 2017). How transcription factors partner with one another to regulate expression of genes specifying different cell states is critical to this process. *Xenopus* has been used as an experimental model for the elucidation of germ layer specification (reviewed by Cao, 2015; Kiecker et al., 2016) and the maternal-to-zygotic transition (reviewed by Jukam et al., 2017).

Wnt signaling, mediated by the intracellular transducer β-catenin (Ctnnb1), plays drastically different roles before and after the maternal-to-zygotic transition (reviewed by Hikasa and Sokol, 2013; Zylkiewicz et al., 2014). Wnt/β-catenin functions in a regulatory switch mechanism to specify very different cell fates within a narrow window of developmental time. First, maternal Wnt signaling-regulated β-catenin protein controls subsequent expression of direct target genes (Blythe et al., 2010), including *siamois* (Brannon et al., 1997; Laurent et al., 1997) and *nodal3* (McKendry et al., 1997; Smith et al., 1995), by the midblastula stage. These genes are among the earliest zygotically expressed factors (Collart et al., 2014; Gentsch et al., 2019a, 2019b; Owens et al., 2016; Skirkanich et al., 2011; Tan et al., 2013; Yang et al., 2002) and function to establish dorsal embryonic cell fates (e.g., Ding et al., 2017; Kessler, 1997; Smith et al., 1995) together with subsequently expressed dorsal genes, such as *goosecoid* (*gsc*) and *noggin* (*nog*) (Ding et al., 2017; Wessely et al., 2001). Within an hour, zygotic Wnt8a signaling functions to regulate a radically different set of direct target genes (Christian et al., 1991; Ding et al., 2017; Hamilton et al., 2001; Hoppler et al., 1996; Nakamura et al., 2016), which then function to restrict dorsal and promote lateral and ventral cell fates (Christian and Moon, 1993; Hoppler et al., 1996). Context-specific direct Wnt/β-catenin target gene expression during these early gastrula stages is defined by co-regulation with Bmp and Fgf signaling (Nakamura et al., 2016); i.e., zygotically expressed Wnt8a regulates β-catenin recruitment to cis-regulatory sequences, whereas target gene transcription is determined by Bmp (Hoppler and Moon, 1998) or Fgf signaling (see also Kjolby et al., 2019). Remarkably, regulation by Bmp and Fgf signaling occurs independently of Wnt8a-regulated β-catenin recruitment to target loci (reviewed by Nakamura and Hoppler, 2017; and Ramakrishnan and Cadigan, 2017).

Here we investigate the regulation of direct gene targets of maternal Wnt/β-catenin signaling at the genome-wide level. Different from the later zygotic direct Wnt8a/β-catenin target genes, we find these direct maternal targets are co-regulated by Foxh1-mediated Nodal/Tgfβ signaling. Our results further define two distinct classes of direct maternal Wnt target genes, which differ both in persistence of β-catenin association and temporal expression, with early genes involved in controlling expression of later ones in an apparent feedforward regulatory loop.

## Results

### Defining the Maternal Wnt/β-Catenin-Regulated Transcriptome

**Figure 1:**
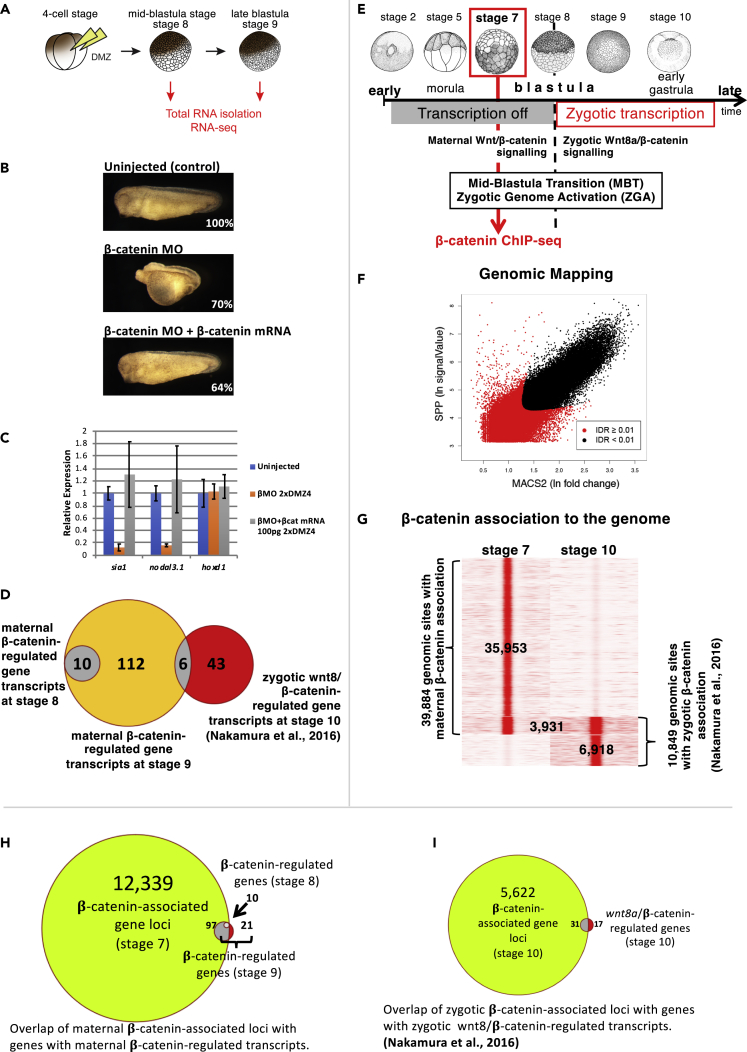
Identification of Maternal Wnt/β-Catenin Target Genes by Combining Transcriptomics (RNA-Seq Analysis) and β-Catenin-Association to Genomic Sequences (β-Catenin ChIP-Seq Analysis) (A) Experimental design of transcriptomics analysis involved targeted injection into the prospective dorsal mesoderm (dorsal marginal zone) of four-cell-stage morula embryos with β-catenin Morpholino (MO, to knock down endogenous β-catenin protein expression) and (where indicated) with β-catenin mRNA (to experimentally rescue maternal Wnt/β-catenin signaling), with RNA expression subsequently sampled at the onset of ZGA (stage 8) and 1 h later (st. 9; with validated triplicate samples [see (C)] used for RNA-seq analysis). (B) Experimental conditions were initially optimized by monitoring expected morphological changes caused by β-catenin knockdown and maternal β-catenin rescue (shown phenotypes are representative of five independent experiments scoring a total of 157, 72, and 174 embryos, respectively, from top to bottom). (C) Extracted RNA samples were validated by monitoring the expected reduced and recovered expression of known maternal Wnt/β-catenin target genes (*sia1*, *nodal3*.*1*; and a zygotic Wnt8/β-catenin target [*hoxd1*] as a negative control) by qPCR following knockdown and rescue, respectively (error bar represents standard deviation from two independent biological experiments with three technical replicates each), before three independent experiments were sequenced. (D) Venn diagram illustrating the number of genes identified (false discovery rate [FDR] <0.05) to be transcriptionally regulated by maternal Wnt/β-catenin signaling at the onset of ZGA (st.8, Table S1A) and 1 h later (st. 9, Table S1B and Figure S1), compared with genes regulated by zygotic Wnt8/β-catenin signaling (st. 10, Table S1C, experimental data from Nakamura et al. [2016], Figure S2); for these two groups of maternal Wnt/β-catenin signaling-regulated genes, also see Figure 2 and Table S1D. (E) Experimental design of β-catenin ChIP-seq analysis at early blastula stage (st.7; before the onset of ZGA) involved pooling of many embryos, since there are fewer cells at early embryonic stages, and therefore fewer nuclei and less DNA. (F) Genomic mapping of β-catenin ChIP-seq experiment with two independent software tools (see Transparent Methods for detail) identifying 39,884 β-catenin-associated genomic locations, near to 12,436 annotated genes. (G) Comparing β-catenin association to the genome before (st.7) and after the onset of ZGA (in the early gastrula, st.10, experimental data from Nakamura et al. [2016]) reveals 3,931 shared β-catenin-associated locations (i.e., same genomic location occupied at st. 7 by maternal β-catenin and at st. 10 by zygotic β-catenin), exclusively maternal β-catenin-associated (35,953), and exclusively zygotic β-catenin-associated locations (6,918). (H) Identification of direct maternal wnt/β-catenin target genes from overlap between maternal β-catenin-associated loci (F and G) with genes with maternal β-catenin-regulated transcripts (D) at stage 8 (first surge of gene expression) and at stage 9 (second surge of gene expression) (Table S1E). (I) As comparison, identification of zygotic Wnt8a/β-catenin targets from overlap between zygotic β-catenin-associated loci with genes with zygotic Wnt8/β-catenin-regulated transcripts (Table S1F, experimental data from Nakamura et al. [2016]).

To identify genes regulated by maternal Wnt/β-catenin signaling, we used an experimental design involving not only knockdown of endogenous β-catenin expression (Ding et al., 2017; Gentsch et al., 2019a, 2019b) but also rescue with re-instated β-catenin expression (Figures 1A and 1B). We validated experimental samples using RT-qPCR by monitoring expected changes in expression of known marker genes (i.e., *sia1* and *nodal3*) at midblastula stage (Figure 1C).

Samples were then processed for RNA sequencing (RNA-seq) analysis. Remarkably, our knockdown and rescue experimental design identify transcripts of only ten genes significantly regulated by maternal Wnt signaling at the early onset of ZGA (midblastula). All turn out to encode paralogs of *siamois* or *nodal3*, *5*, *6* (Table S1A). Since other known Wnt-regulated, dorsally expressed genes (such as *gsc*, *nog*, *chrd*, and *fst1*, e.g., Wessely et al. [2001] and Ding et al. [2017]) were not among these genes, we analyzed from the same experiment samples collected later, 1 h after the initial onset of ZGA (late blastula, Table S1B). At this stage, we find transcripts of 128 genes significantly regulated by maternal Wnt signaling, among them the ten already identified at the initial onset of ZGA (Figure 1D).

**Figure 2:**
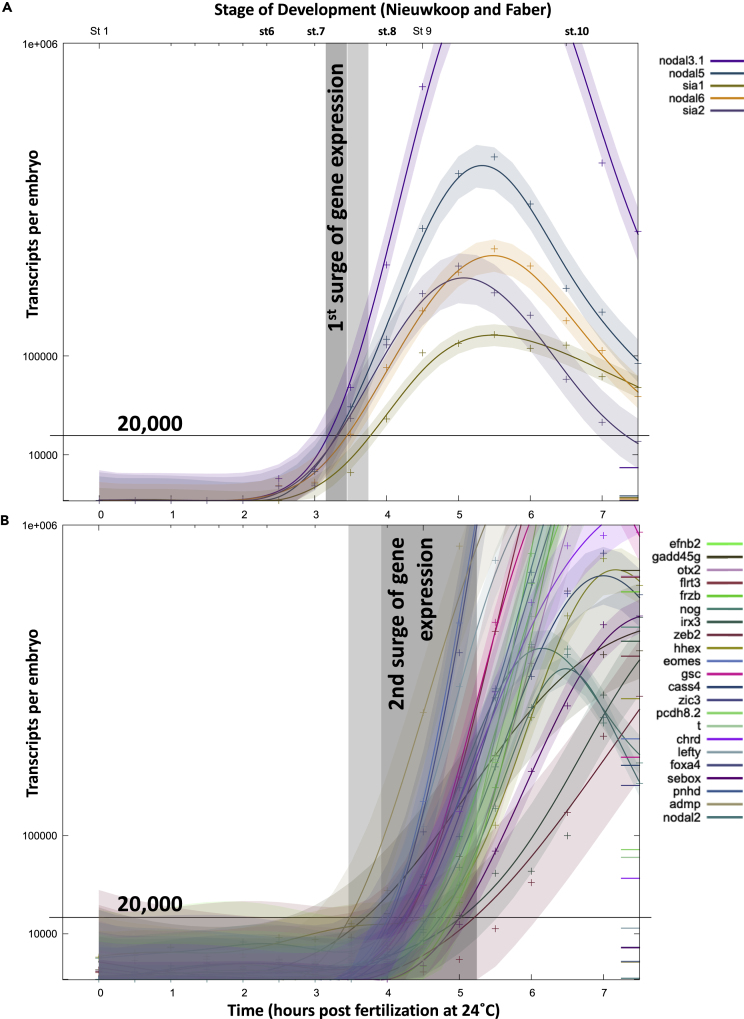
Two Surges of Maternal Wnt/β-Catenin Target Gene Expression (A) First surge of maternal Wnt/β-catenin-regulated gene expression initiates between stage 7 and stage 7.5 (gray box), although sia1 is slightly delayed (lighter gray box) relative to the other genes in this class (sia2, nodal3.1, nodal 5, nodal 6). (B) Second surge of maternal Wnt/β-catenin-regulated gene expression initiates between stage 8 and stage 9.5 (gray box), although admp and gadd45g are slightly earlier (lighter gray box) than the other genes in this class (e.g., eomes, gsc, chrd, frzb, noggin, nodal 2, and others as indicated). Data were mined from Owens et al. (2016) using the online tool http://genomics.crick.ac.uk/cgi-bin/profile-search.exe?dbe=http&dbs=INFO-PUBLIC&uid=guest&species=Xt&profiles=KBAP&src=search&tgt=main&menu=main_images&option=images&dataset=KBAP&project_key=0&version=0. The graphs shown are framed between zero and 1 million transcripts per embryo and between fertilization and stage 10.25. Of the ten maternal Wnt/β-catenin-regulated genes identified as a first surge of expression in our analysis (using version 9 of the *Xenopus tropicalis* genome assembly, Figure 1D and Table S1A), the transcriptomics data from Owens et al. (2016, analyzed using version 7) contained information for five (see Table S1D), whereas of the 112 maternal Wnt/β-catenin-regulated genes expressed exclusively as part of the second surge of expression (118 minus the 10 genes already expressed from the first surge), 22 were used in this analysis both because transcriptomics data from Owens et al. (2016) were available and induction could be defined between low initial gene expression (less than 10k transcripts per embryo before st.6) and increased expression (more than 100k by stage 10, see Table S1D). The gray boxes indicate the first (in A) and second surge (in B) of expression, defined by 20,000 transcripts per embryo in the transcriptomics data from Owens et al. (2016).

Our transcriptomics analysis therefore reveals two groups of maternal Wnt-regulated genes: the first surge of expression includes what has previously been described as “pre-MBT” transcription (Yang et al., 2002), and then the second surge of gene expression concurs with more general onset of ZGA, including dorsally expressed genes, including *gsc* and *nog*, which had previously been shown to be Wnt regulated (Ding et al., 2017; Wessely et al., 2001). These two distinct surges of gene expression related to these two groups can also be seen in the staged transcriptomics data by Owens et al. (2016) (Figures 2A and 2B).

### β-Catenin Protein Associates with Genomic Loci Prior to ZGA

In order to identify direct Wnt/β-catenin target genes among maternal Wnt-regulated genes, we embarked on β-catenin chromatin immunoprecipitation sequencing (ChIP-seq) analysis. β-Catenin indirectly associates with genomic DNA sequences by binding sequence-specific DNA binding transcription factors, principally of the LEF/TCF protein family (e.g., Nakamura et al., 2016). We performed β-catenin ChIP-seq analysis in the early blastula, revealing β-catenin association with 39,884 specific genomic sites (Figures 1E–1G), which can be bioinformatically assigned to 12,339 annotated genes (Figure 1H).

We then compared this genome association of maternal Wnt-regulated β-catenin before with the genome association of zygotic Wnt-regulated β-catenin well after ZGA (early gastrula, Nakamura et al., 2016, reanalyzed the same way as the new data). This comparison revealed 35,953 exclusively maternal β-catenin bindings sites (peaks) distinct from 6,918 exclusively zygotic binding sites, with 3,931 overlapping (i.e., loci associated with β-catenin before and after ZGA, Figure 1G).

When comparing our transcriptomics with our genome association results, we find that all ten maternal Wnt/β-catenin-regulated genes in the first group, i.e., with an early surge of expression, have nearby maternal β-catenin association, indicating that, as expected, they are all direct target genes (cf. Blythe et al., 2010), as are 82% of the second group of maternal Wnt/β-catenin-regulated genes with a later surge of gene expression (Figure 1H, compare with direct Wnt8a/β-catenin target genes at gastrulation, Figure 1I). All maternal Wnt-regulated genes expressed in the first group show β-catenin binding in the early blastula stage ChIP-seq data, but significantly, not at early gastrula stage (Figures 1G, S3A, and S3B). In contrast, maternal Wnt-regulated genes expressed in the second surge show β-catenin binding both before and after ZGA (Figures 1G, S3C, and S3D; data from Nakamura et al. [2016]).

Both transcriptomics and β-catenin ChIP-seq analyses therefore independently identify the same two distinct classes of direct maternal Wnt/β-catenin target genes in the early embryo.

### Coherent Feedforward Regulation of Direct Maternal Wnt/β-Catenin Target Genes of the Second Class by Gene Products of the First

**Figure 3:**
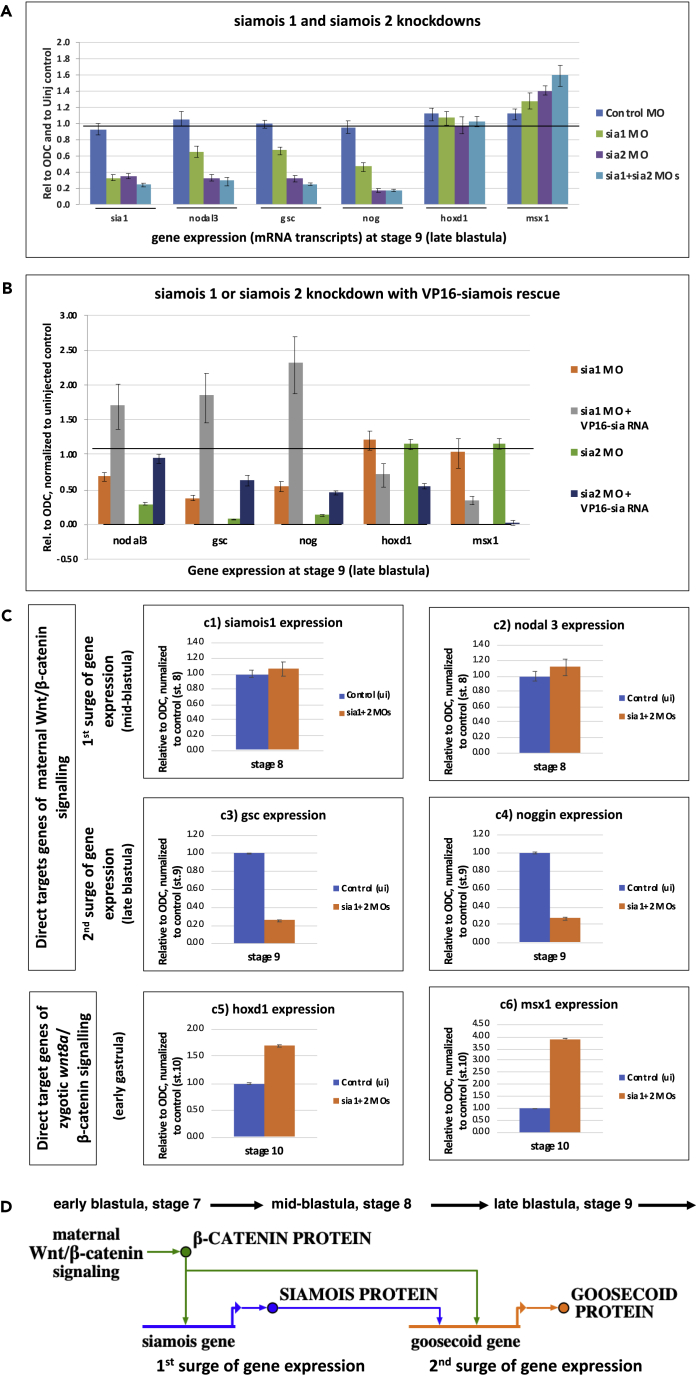
Coherent Feedforward Regulation of Maternal Wnt/β-Catenin Target Genes in Late Blastula (A) *siamois1*, *siamois2* (also known as *twin*) and double Morpholino (MO) knockdown causes reduced expression of maternal Wnt/β-catenin target genes (at late blastula stage 9) (see also Figures S4A–S4F). (B) Rescue of *siamois1* and *siamois2* knockdown with constitutively activating siamois construct (which is not targeted by either MO) re-instates expression of maternal Wnt/β-catenin target genes (*nodal3*, *goosecoid*, *noggin*). (C) Stage-specific sampling of gene expression reveals that direct maternal Wnt/β-catenin target genes of the first class (*siamois1* and *nodal3*) remain unaffected (st. 8), whereas expression of direct maternal Wnt/β-catenin target genes of the second class (*goosecoid*, *noggin*) is reduced (st.9). Also note that expression of zygotic Wnt8/β-catenin target genes (*hoxd1*, *msx1*) is not reduced but may be increased presumably owing to indirect mechanisms. (D) Coherent feedforward regulation of some direct maternal Wnt/β-catenin target genes of the second class (e.g., *goosecoid*) involves *siamois* genes, which are among direct maternal Wnt/β-catenin target genes of the first class. Control Morpholino (control MO-injected embryos); Uninjected Control (uninjected embryos); sia1 MO, sia2 MO (embryos injected with Morpholino targeting *siamois1* or *siamois2* [also known as twin], respectively; VP16-sia RNA (*Xenopus tropicalis* embryos injected with *Xenopus laevis* constitutively active siamois mRNA [Kessler, 1997]). Data are from one representative of three independent experiments; error bars represent mean ± SEM of three technical replicates with p ≤ 0.05.

What could account for the temporal difference in timing of expression between these two classes of direct maternal Wnt/β-catenin target genes? We wondered whether products of direct maternal Wnt target genes expressed as part of the first surge might be required for regulation of direct maternal Wnt targets in the second surge, since some of the genes in this second class of direct Wnt target genes had previously been shown to be regulated by *siamois* (e.g., Bae et al., 2011; Carnac et al., 1996). MO knockdown of *sia1* and *sia2* indeed results in reduced expression of some direct maternal Wnt target genes of the second class (Figure 3A, as also previously shown in Bae et al. [2011]), which is reinstated by rescuing Sia activity (Figure 3B), whereas the earlier surge of expression of the first class of direct target genes (in midblastula), such as *sia1* itself and *nodal3*, is not affected (Figure 3C).

These results support our hypothesis that maternal Wnt/β-catenin regulates these two classes differently, with second-class genes additionally requiring products of the first class. This suggests that a coherent feedforward regulatory mechanism (Figure 3D) promotes expression of direct Wnt target genes of the second class.

### Foxh1/Nodal Signaling Is Required for Context-Specific Regulation of Direct Maternal Wnt/β-Catenin Target Genes in the Early Embryo

Previously we had found that β-catenin association with cis-regulatory sequences is insufficient for transcriptional regulation of Wnt8a/β-catenin target genes in specification of ventral tissue in the early gastrula (Nakamura et al., 2016; Nakamura and Hoppler, 2017). Bmp or Fgf signaling was identified as critical for the context-specific expression of these zygotic Wnt8a/β-catenin target genes (Hoppler and Moon, 1998; Kjolby et al., 2019; Nakamura et al., 2016). Here, we aimed to determine what context-determining mechanism is involved in regulating direct maternal Wnt/β-catenin target genes, which are expressed earlier in the dorsal marginal zone of blastula-stage embryos.

We used *de novo* motif analysis to identify shared cis-regulatory sequences suggesting transcription factor binding sites among direct maternal Wnt/β-catenin target genes. As expected, these genes share sequences for LEF/TCF-binding sites (also known as WRE, reviewed by Ramakrishnan and Cadigan, 2017). Importantly, in addition, they also harbor motifs matching the consensus binding site for Foxh1 (Table 1). Motif analysis of zygotic Wnt8a/β-catenin target sequences had not identified Foxh1 consensus binding sequences (Nakamura et al., 2016). This difference suggests that Foxh1 plays a context-determining role in selecting which of the many maternal β-catenin-bound genes are transcriptionally regulated by maternal Wnt/β-catenin.

**Table 1:** *De Novo* Motif Analysis of β-Catenin-Associated Cis-regulatory Sequences

A: *De Novo* Motif Analysis First Surge Genes					B: *De Novo* Motif Analysis Second Surge Genes				
Rank	Discovered Motif	Best Match	p Value	%	Rank	Discovered Motif	Best Match	p Value	%
1		TCF7L2	110^−20^	26	1		FOXH1	1 × 10^−99^	49
2		FOXH1	1 × 10^−19^	32	2		Tbx21-like T-box	1 × 10^−41^	14
3		LEF1	1 × 10^−18^	35	3		Helix-turnhelix (homeobox?)	1 × 10^−28^	5
4		Pan/dTCF	1 × 10^−18^	23	4		TCF7L2	1 × 10^−28^	18
5		Pan/dTCF	1 × 10^−18^	16	5		C2H2 zinc finger	1 × 10^−26^	4
6		C4 zinc finger (GATA?)	1 × 10^−16^	16	6		ROX1-like HMGbox	1 × 10^−25^	7
7		Zinc finger	1 × 10^−15^	23	7		NFkB-like	1 × 10^−25^	42
8		SOX	1 × 10^−14^	13	8		TDA9-like zinc finger	1 × 10^−24^	6
9		Meis1	1 × 10^−13^	42	9		MBP1-like helix-turnhelix	1 × 10^−22^	15
10		TOD6-like	1 × 10^−13^	26	10		NHP10-like HMG box	1 × 10^−14^	7

*De novo* motif analysis of sequences surrounding β-catenin-associated locations (at early blastula st.7) in maternal Wnt/β-catenin-regulated genes at the onset of zygotic transcription (ZGA) (Table 1A, mid-blastula st.8, note Tcf/Lef and Foxh1 consensus motifs) and approximately 1 h later (Table 1B, late blastula st. 9, note Foxh1 and Tcf/Lef consensus binding motifs).

**Figure 4:**
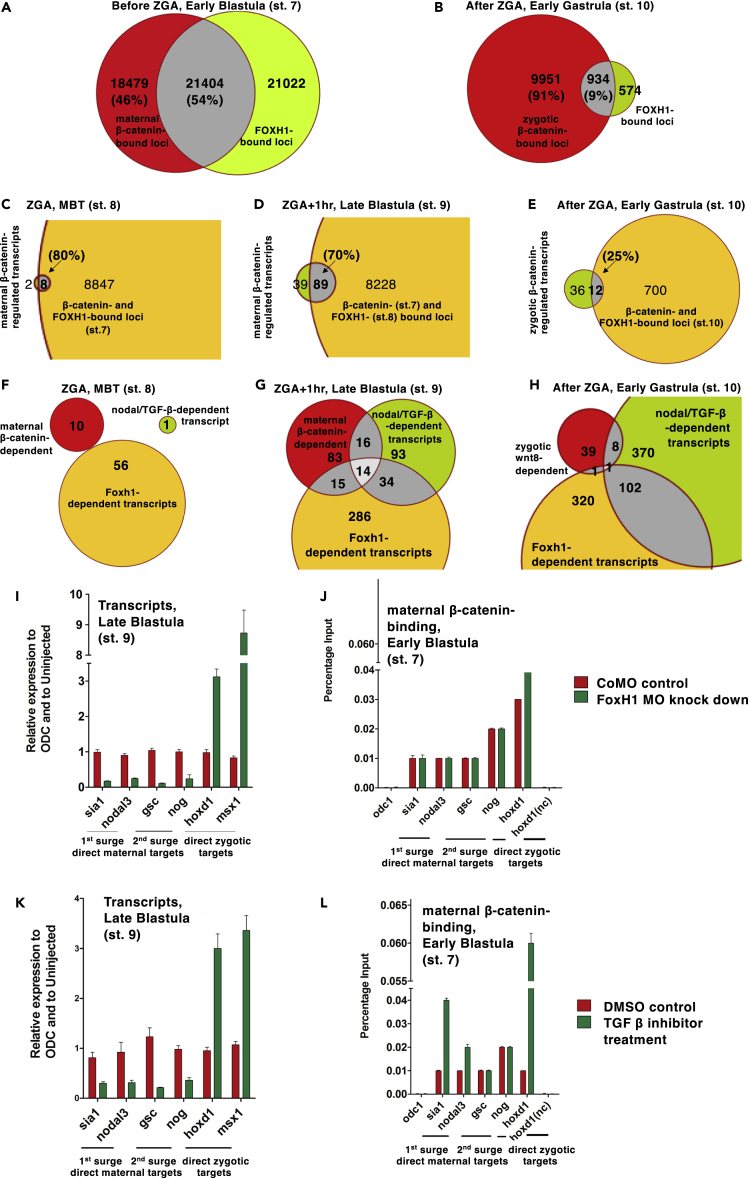
Foxh1/Nodal Signaling Is Required for Expression of Maternal Wnt Target Genes but Not for β-Catenin Recruitment (A and B) Bioinformatics analysis of overlap between genomic loci with maternal β-catenin and Foxh1 association before the onset of zygotic transcription (Zygotic Gene Activation, ZGA) (A, st.7, Early Blastula); and after (B, st.10, Early Gastrula). Note considerable overlap before the onset of ZGA (A, compared with B; also see Tables S2A and S2B). (C–E) Overlap between Wnt/β-catenin-regulated gene loci and genomic loci sharing β-catenin and Foxh1 association; comparing (C) maternal Wnt/β-catenin-regulated gene loci at the onset of ZGA (st. 8, midblastula) with shared β-catenin/Foxh1-associated loci just before the onset of ZGA (st. 7, early blastula); (D) maternal Wnt/β-catenin-regulated gene loci after the onset of ZGA (st. 9, late blastula) with shared β-catenin/Foxh1 loci at the onset of ZGA (comparing β-catenin-associated loci at st.7 with Foxh1-associated loci at st. 8, midblastula); and (E) Wnt8a/β-catenin-regulated gene loci with β-catenin/Foxh1 loci at early gastrulation (st. 10, early gastrula). Note correlation between maternal Wnt/β-catenin-regulated gene loci and corresponding β-catenin/Foxh1 loci (80% and 70%, respectively) of maternal Wnt/β-catenin signaling-regulated gene loci expressed in the first surge (C) and in the second surge of gene expression (D), compared with less than 25% of zygotic Wnt8a/β-catenin signaling-regulated gene loci in (E) (see also Tables S2C–S2E). (F–L) Overlap between Wnt/β-catenin-regulated genes and transcripts reduced in a zygotic Foxh1 morpholino knockdown, and following pharmacological inhibition of Nodal/Tgfβ signaling (with SB431542, SB), at the onset of ZGA (F, st.8, midblastula), 1 h later (G, st. 9, late blastula), and during early gastrulation (H, st. 10, early gastrula) (see also Tables S2F–S2H). Note correlation between maternal Wnt/β-catenin-regulated genes and those reduced in Foxh1 knockdown and with inhibited Nodal signaling (G), compared with zygotic Wnt8a/β-catenin-regulated genes at st.10 in (H). Absence of overlap when analyzed at midblastula stage (st.8 in [F]) is likely due to maternal rescue, i.e., maternal FOXH1 protein not affected by Foxh1 morpholino knockdown. Foxh1 Morpholino knockdown (I and J) and pharmacological inhibition of Nodal signaling with SB431542 (K and L) cause reduced gene expression of representative maternal Wnt/β-catenin target genes (I and K; analyzed with qRT-PCR, see also Figures S4G, S4H, S4J, and S4K) but does not cause reduced β-catenin association at these loci (J and L; analyzed with β-catenin ChIP-qPCR, see also Figures S4I and S4L). Data are from one representative of three independent experiments; error bars represent propagation error of three technical replicates with p ≤ 0.05.

We used Foxh1 ChIP-seq data (as in Charney et al., 2017; Chiu et al., 2014) to explore this hypothesis. Comparing β-catenin-bound with Foxh1-bound regions reveals a substantial (54%) correlation before ZGA (Figure 4A), but not thereafter (9%) (Figure 4B). A similar finding of enrichment for Foxh1 was recently reported by Gentsch et al. (2019b). There is also a strong correlation between these genomic loci that share both β-catenin and Foxh1 association with the maternal Wnt/β-catenin targets that we had identified above (Figures 4C and 4D, 80% and 70%, respectively; but less so [25%] with zygotic Wnt8a/β-catenin targets Figure 4E). We also compared Wnt/β-catenin-regulated genes with altered gene expression in an MO-mediated knockdown of *foxh1* function (Figures 4F–4H, see Transparent Methods). There is higher correlation in late blastula (Figure 4G, i.e., 23% of maternal Wnt/β-catenin-regulated transcriptome) than later in early gastrula (Figure 4H, i.e., 5% of zygotic Wnt8a/β-catenin-regulated transcriptome). This analysis correlates context-specific regulation of maternal Wnt/β-catenin target genes with a requirement for Foxh1 function.

Since Foxh1 function in the early embryo mediates embryonic Nodal/Tgfβ signaling (Chen et al., 1996; Chiu et al., 2014; Hill, 2018), we compared the maternal Wnt/β-catenin-regulated transcriptome directly with transcripts reduced after treatment with a pharmacological Nodal/Tgfβ signaling inhibitor, SB431542 (Figures 4F–4H). We expected to find such a correlation, since cooperative regulation by Nodal/Tgfβ and Wnt/β-catenin-signaling had been demonstrated for some of the genes identified here as direct maternal Wnt/β-catenin target genes of class 1 (*sia*) and class 2 (*gsc*, *chrd*) (Crease et al., 1998; Nishita et al., 2000). As with Foxh1 above, there is indeed correlation between Nodal//Tgfβ- and maternal Wnt/β-catenin-regulated genes (Figure 4G, 23%), which, however, is only slightly higher than with zygotic Wnt8a/β-catenin-regulated genes (Figure 4H, 18%), which may reflect a mostly Foxh1-independent role for Nodal/Tgfβ signaling in control of zygotic Wnt8a/β-catenin-regulated genes (e.g., Charney et al., 2017; Coda et al., 2017; Germain et al., 2000; Kunwar et al., 2003). In conclusion, maternal Wnt/β-catenin signaling target genes could be co-regulated by Foxh1/Tgfβ.

### β-Catenin Association with Target Genes Is Independent of Foxh1/Nodal Signaling

We directly validated the requirement of Foxh1 function and Nodal/Tgfβ signaling activity for regulation of direct maternal Wnt/β-catenin target genes using a *foxh1* knockdown and a pharmacological Nodal/Tgfβ signaling inhibitor (Chiu et al., 2014). *foxh1* knockdown (Figure 4I) and inhibition of Nodal/Tgfβ signaling (Figure 4K) caused reduced expression of direct maternal Wnt/β-catenin target genes at the late blastula stage, both those of the first class (*sia1*, *nodal3*) and of the second class (*gsc*, *nog*).

Since we had previously shown that loss of context-defining Bmp or Fgf signaling had no effect on β-catenin recruitment to zygotic Wnt8a/β-catenin target loci (Nakamura et al., 2016), we tested here whether Foxh1/Tgfβ signaling could influence β-catenin recruitment to relevant WREs in blastula stage embryo, using β-catenin ChIP-qPCR. No reduction of β-catenin association at maternal Wnt/β-catenin target loci is detected when Foxh1 function (Figure 4J) or Nodal/Tgfβ signaling is inhibited (Figure 4L). These results demonstrate that Foxh1 function and Nodal/Tgfβ signaling are required independently of Wnt-regulated β-catenin association at maternal Wnt/β-catenin target loci for their context-specific transcriptional expression.

## Discussion

Initially two kinds of direct Wnt/β-catenin target genes were expected in the early embryo (reviewed by Zylkiewicz et al. [2014], Nakamura and Hoppler [2017] and Esmaeili et al. [2020]): direct maternal Wnt/β-catenin target genes, such as *sia1* and *nodal3* (involved in dorsal specification), and direct zygotic Wnt8a/β-catenin target genes, such as *hoxd1* and *ventx1* (involved in ventral/lateral specification). Our previous analysis of direct zygotic Wnt8a/β-catenin target genes had revealed at least two contexts (Bmp-regulated and Fgf-regulated contexts, Nakamura et al., 2016). Here, we describe a much greater developmental complexity of direct maternal Wnt/β-catenin target genes, implicating an additionally dorsally expressed class of genes, expression of some of which were known to be influenced by Wnt signaling (e.g., Ding et al., 2017; Wessely et al., 2001). These two classes of direct maternal Wnt/β-catenin target genes can be defined both by their timing of gene expression and by their dynamics of β-catenin-association with respective genomic loci.

Yet our analysis discovers a shared Foxh1- and Nodal/Tgfβ signaling-dependent context-defining mechanism for both the first and second class of direct maternal Wnt/β-catenin target genes. Cooperative regulation of early dorsal embryonic development by Nodal/Tgfβ and Wnt/β-catenin signaling is deeply conserved among vertebrates and even with closely related invertebrate chordates (Kozmikova and Kozmik, 2020). Thus, maternal Wnt/β-catenin regulation of direct transcriptional targets occurs in a different co-regulatory context (i.e., Foxh1 and Nodal/Tgfβ) than for direct zygotic Wnt8a/β-catenin targets (i.e., Bmp or Fgf). Importantly, Wnt signaling regulates β-catenin association with direct Wnt/β-catenin target loci in all these different contexts independently of any of those various context-defining co-regulatory mechanisms, which in turn only regulate the expression of, not β-catenin-association with, these Wnt target genes.

However, the first class of direct maternal Wnt/β-catenin target genes lose β-catenin association by gastrulation, precisely when chromatin accessibility at such loci is found to be restricted (Esmaeili et al., 2020). Developmental competence of direct target genes to respond to Wnt/β-catenin signaling in a context-specific way is therefore likely to be regulated not only by combinatorial signaling as highlighted here but also by developmentally regulated chromatin modification, which we have not further explored (see also Hontelez et al., 2015).

Wnt-activated nuclear β-catenin associates widely with chromatin across the genome, including to many loci that are not expressed at the stages analyzed (see also Nakamura et al., 2016). It is likely that such extra binding, which is not regulating stage-specific transcription nearby, may function as a buffering mechanism to fine-tune the response and prevent inadvertent promotion of transcription (as initially proposed for transcription factors by Lin and Riggs [1975] and discussed in the context of Wnt/β-catenin signaling in Nakamura and Hoppler [2017]).

The two classes of direct maternal Wnt/β-catenin target genes differ in that the specific context-defining mechanism controlling gene expression of the second class includes a coherent feedforward mechanism involving an additional input from products of genes of the first class of direct maternal Wnt/β-catenin targets (Figure 3D). Such a coherent feedforward regulatory network motif was shown to serve as a persistence detector (a so-called sign-sensitive delay element, e.g., Mangan and Alon, 2003), suggesting here that only persistent maternal Wnt/β-catenin signaling will promote second class target gene expression and subsequent dorsal axis development. Additional gene regulatory mechanisms are not ruled out, particularly since additional consensus transcription factor binding motifs were discovered in relevant β-catenin-associated genomic DNA sequences (Table 1, e.g., Sox3, see also Doumpas et al., 2019; Gentsch et al., 2019b; Kormish et al., 2010; Zhang et al., 2003).

The concepts we uncover about regulation of direct Wnt/β-catenin target genes in the early *Xenopus* embryo provides a general novel paradigm for the role of context in Wnt target gene regulation in other developmental settings and in human disease, such as cancer (e.g., Koval and Katanaev, 2018; Madan et al., 2018).

### Limitations of the Study

The concept of feedforward regulation emphasized here implies redundancy in gene regulation, which may have evolved for improved robustness. This redundancy, by definition, makes it difficult to disentangle direct from indirect inputs and demonstrate that both are required independently for gene activation.

### Resource Availability

#### Lead Contact

Stefan Hoppler (s.p.hoppler@abdn.ac.uk).

#### Materials Availability

Requests for materials and reagents should be directed to the Lead Contact.

#### Data Availability

Raw sequencing data generated for this study have been deposited in the ArrayExpress database at EMBL-EBI under the accession number E-MTAB-8555 (http://www.ebi.ac.uk/arrayexpress/experiments/E-MTAB-8555).

Previously published datasets used in this study are available from Gene Expression Omnibus at NCBI under the accession numbers GSE53654, GSE72657, and GSE85273.

## Methods

All methods can be found in the accompanying Transparent Methods supplemental file.

## Supplemental Information

Document S1. Transparent Methods and Figures S1–S4

Table S1: Wnt/β-catenin Target Genes, Related to Figure 1 Sheet A: Genes regulated by maternal Wnt/β-catenin signaling at midblastula (stage 8, see Figure 1D). Sheet B: Genes regulated by maternal Wnt/β-catenin signaling at late blastula (stage 9, see Figure 1D). Sheet C: Genes regulated by zygotic Wnt8a/β-catenin signaling at early gastrula (stage 10, see Figure 1D, data from Nakamura et al. (2016)). Sheet D: Selected gene list for the first and second groups of maternal Wnt/β-catenin signaling-regulated transcripts in developmental series from Owens et al. (2016), see Figure S4). Sheet E: direct maternal β-catenin target genes, determined through overlap between β-catenin-associated loci (stage 7) and maternal Wnt/β-catenin-regulated transcripts (at stages 8 and 9, see Figure 1H). Sheet F: direct zygotic Wnt8a/β-catenin target genes, determined through overlap between β-catenin-associated loci (stage 10) and zygotic Wnt8a/β-catenin-regulated transcripts (see Figure 1I).

Table S2: Correlation between Wnt/β-catenin signaling, Foxh1 and Nodal/TGFβ signaling, Related to Figure 4 Sheet A: List of nearby genes: Correlation between β-catenin-associated and Foxh1-associated loci at early blastula (stage 7, see Figure 4A). Sheet B: List of nearby genes: Correlation between β-catenin-associated and Foxh1-associated loci at early gastrula (stage 10, see Figure 4B). Sheet C: Correlation between direct maternal Wnt/β-catenin target genes expressed as part of the first surge of expression (stage 8) and β-catenin and Foxh1 co-associated loci (stage 7) (see Figure 4C). Sheet D: Correlation between direct maternal Wnt/β-catenin target genes expressed as part of the second surge of expression (stage 9) and β-catenin and Foxh1 co-associated loci (stage 7) (see Figure 4D). Sheet E: Correlation between zygotic Wnt8a/β-catenin target genes (st. 10) and β-catenin and Foxh1 co-associated loci (stage 10) (see Figure 4E) Sheet F: Correlation between maternal Wnt/β-catenin-regulated genes of the first surge of expression, foxh1-dependent and tgfβ signaling-dependent transcripts (stage 8) (see Figure 4F). Sheet G: Correlation between maternal Wnt/β-catenin-regulated genes of the second surge of expression, foxh1-dependent and tgfβ signaling-dependent transcripts (stage 9) (see Figure 4G). Sheet H: Correlation between zygotic Wnt8a/β-catenin-regulated genes, foxh1-dependent and tgfβ signaling-dependent transcripts (stage 10) (see Figure 4H).
