# Supplementary material for: Foxh1/Nodal Defines Context-Specific Direct Maternal Wnt/β-Catenin Target Gene Regulation in Early Development
Source: iScience. 2020 Jun 25;23(7):101314. doi: 10.1016/j.isci.2020.101314 (PMC7347983; doi:10.1016/j.isci.2020.101314)
Supplement: Document S1. Transparent Methods and Figures S1–S4 [file mmc1.pdf]

iScience, Volume *23*

Supplemental Information

Foxh1/Nodal Defines Context-Specific Direct Maternal Wnt/β-Catenin Target Gene Regulation in Early Development

Boni A. Afouda, Yukio Nakamura, Sophie Shaw, Rebekah M. Charney, Kitt D. Paraiso, Ira L. Blitz, Ken W.Y. Cho, and Stefan Hoppler

SUPPLEMENTAL FIGURES

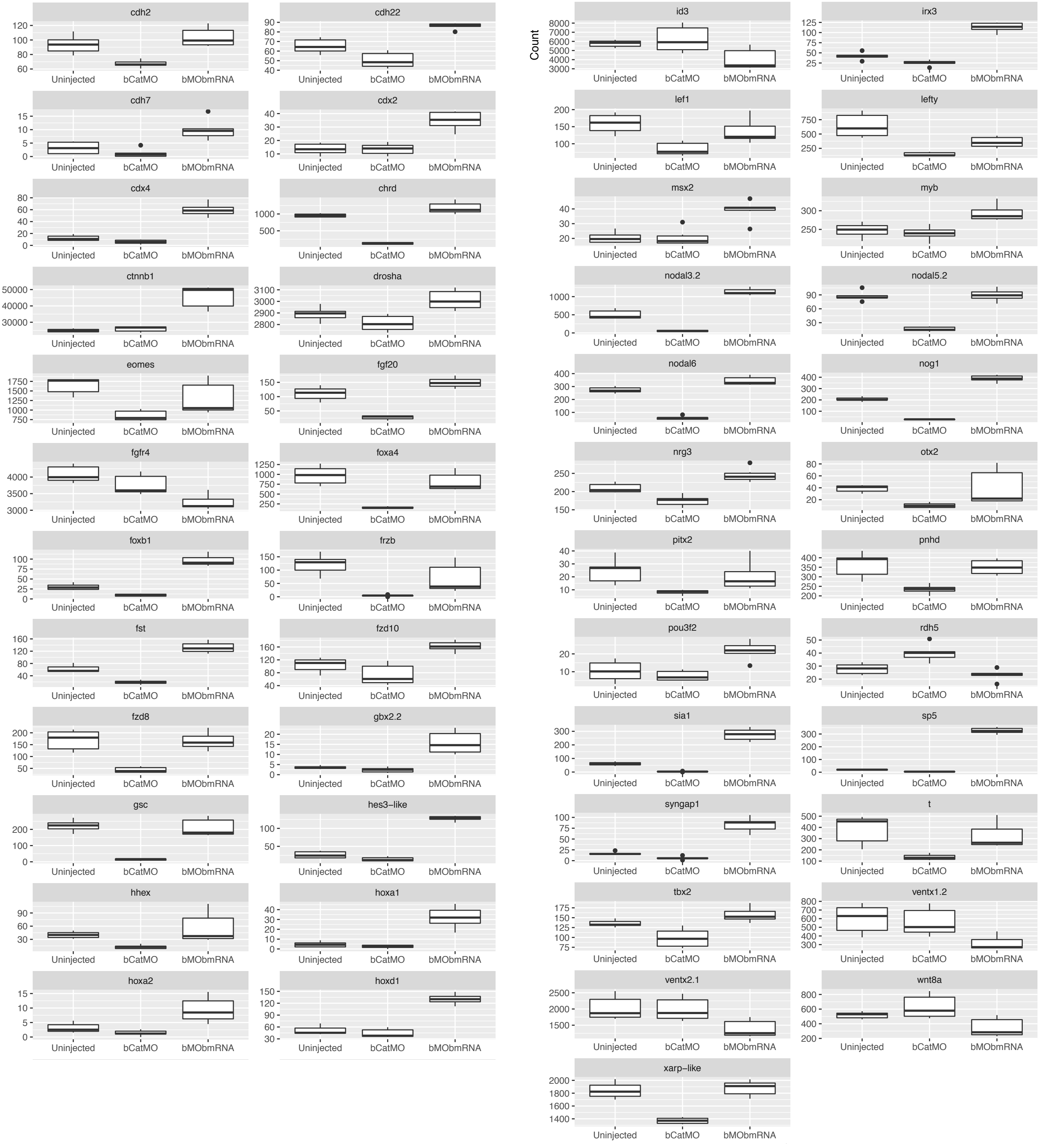

Suppl. Fig.1

Suppl. Figure 1: Transcriptomics of maternal Wnt/β-catenin signaling-regulated genes at stage 9 (late blastula), Related to Figure 1D

Sequence counts for individual genes (in alphabetical order) in the individual samples of the experiment involving control, knock-down and rescue of maternal β-catenin signaling, as indicated.

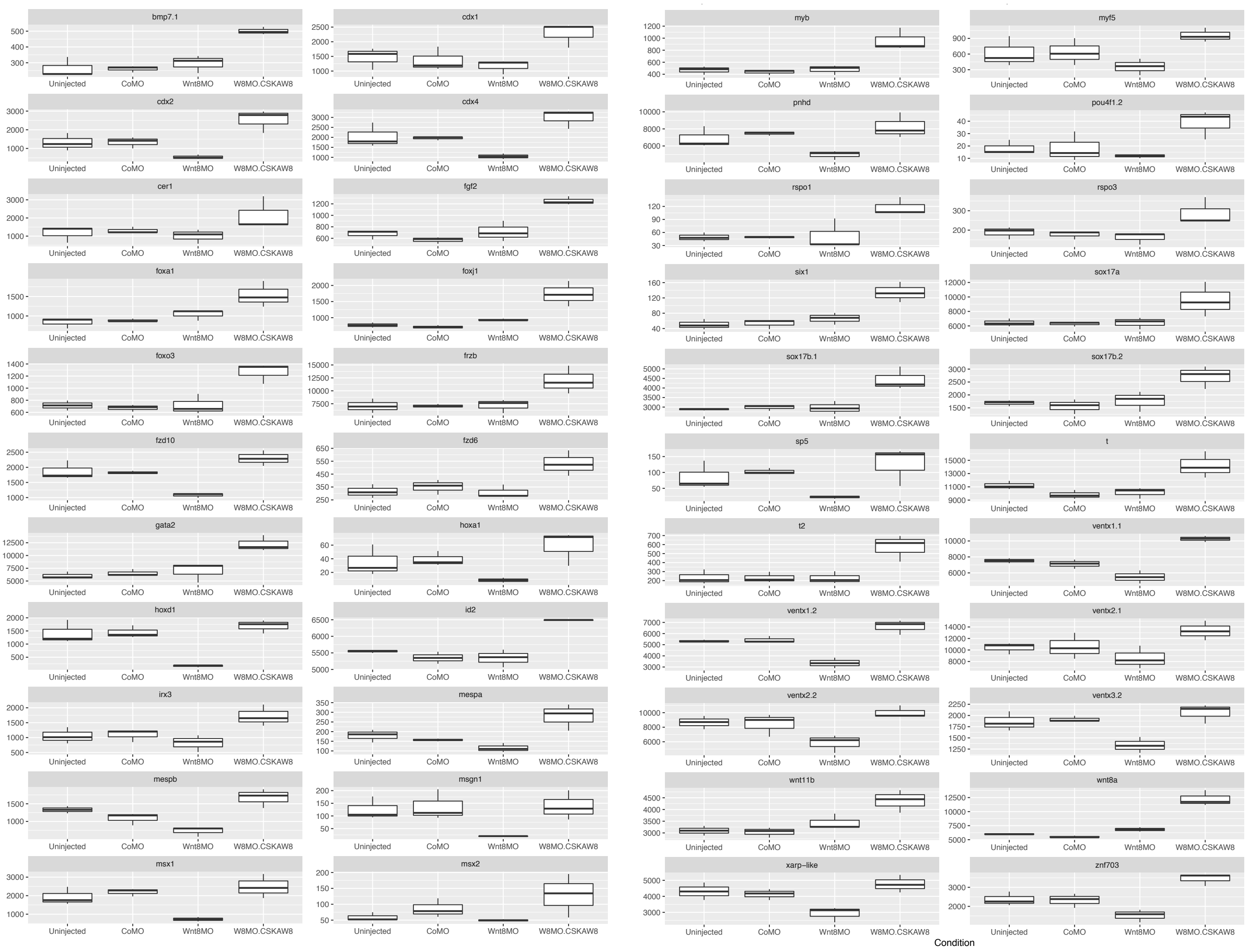
bmp7.1
cdx1
myb
myf5
cdx2
cdx4
pnhd
pou4f1.2
cer1
fgf2
rspo1
rspo3
foxa1
foxj1
six1
sox17a
foxo3
frzb
sox17b.1
sox17b.2
fzd10
fzd6
sp5
t
gata2
hoxa1
t2
ventx1.1
hoxd1
id2
ventx1.2
ventx2.1
irx3
mespa
ventx2.2
ventx3.2
mespb
msgn1
wnt11b
wnt8a
msx1
msx2
xarp-like
znf703
Uninjected
CoMO
Wnt8MO
W8MO.CSKAW8
Condition

Suppl. Fig.2

Suppl. Figure 2: Transcriptomics of zygotic Wnt8a/β-catenin signaling-regulated genes at stage 10 (early gastrula), Related to Figure 1D

Sequence counts for individual genes (in alphabetical order) in the individual samples of the experiment involving controls, knock-down and rescue of zygotic Wnt8a signaling, as indicated. Data from Nakamura et al. (2016) reanalyzed.

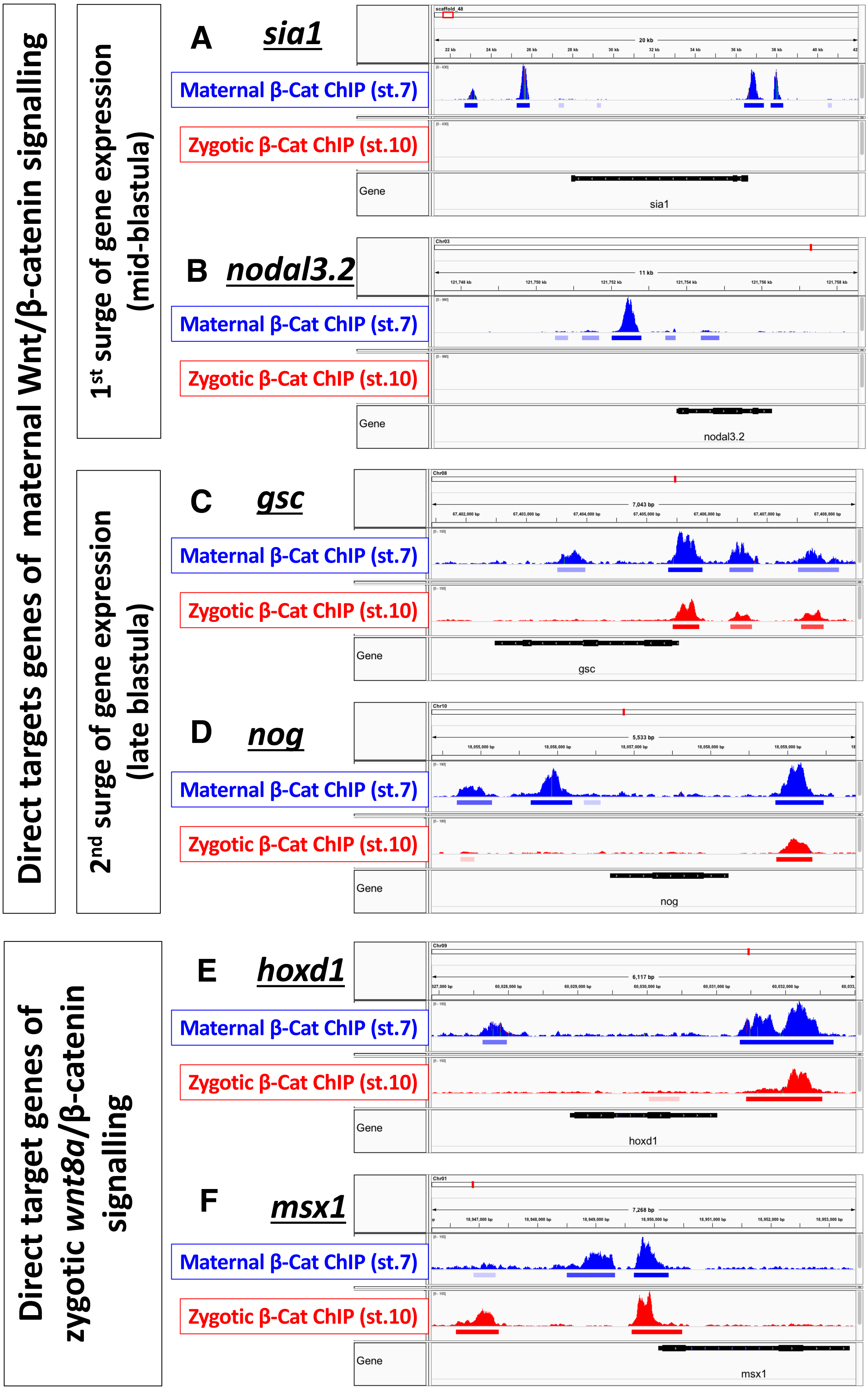
Direct targets genes of maternal Wnt/β-catenin signalling
1st surge of gene expression (mid-blastula)
2nd surge of gene expression (late blastula)
Direct target genes of zygotic wnt8a/β-catenin signalling
A sia1
B nodal3.2
C gsc
D nog
E hoxd1
F msx1
Maternal β-Cat ChIP (st.7)
Zygotic β-Cat ChIP (st.10)
Gene
sia1
nodal3.2
gsc
nog
hoxd1
msx1
scaffold_48
Chr03
Chr08
Chr10
Chr09
Chr01
20 kb
11 kb
7,043 bp
5,533 bp
6,117 bp
7,268 bp

Suppl. Fig. 3

Suppl. Figure 3: Comparing maternal and zygotic β-catenin ChIP-seq signals at example gene loci, Related to Figure 1G

Genome alignment of β-catenin ChIP-seq signals at example gene loci at stage 7 (in blue, to illustrate genome association by β-catenin regulated by maternal Wnt/β-catenin signaling) and stage 10 (in red, to illustrate genome association by β-catenin regulated by zygotic *wnt8*/β-catenin signaling; data from Nakamura et al. (2016). A) *siamois1* (*sia1* gene locus), B) *nodal3.2* gene locus C) *gsc* gene locus D) *noggin* (*nog* gene locus) E) *hoxd1* gene locus and F) *msx1* gene locus.

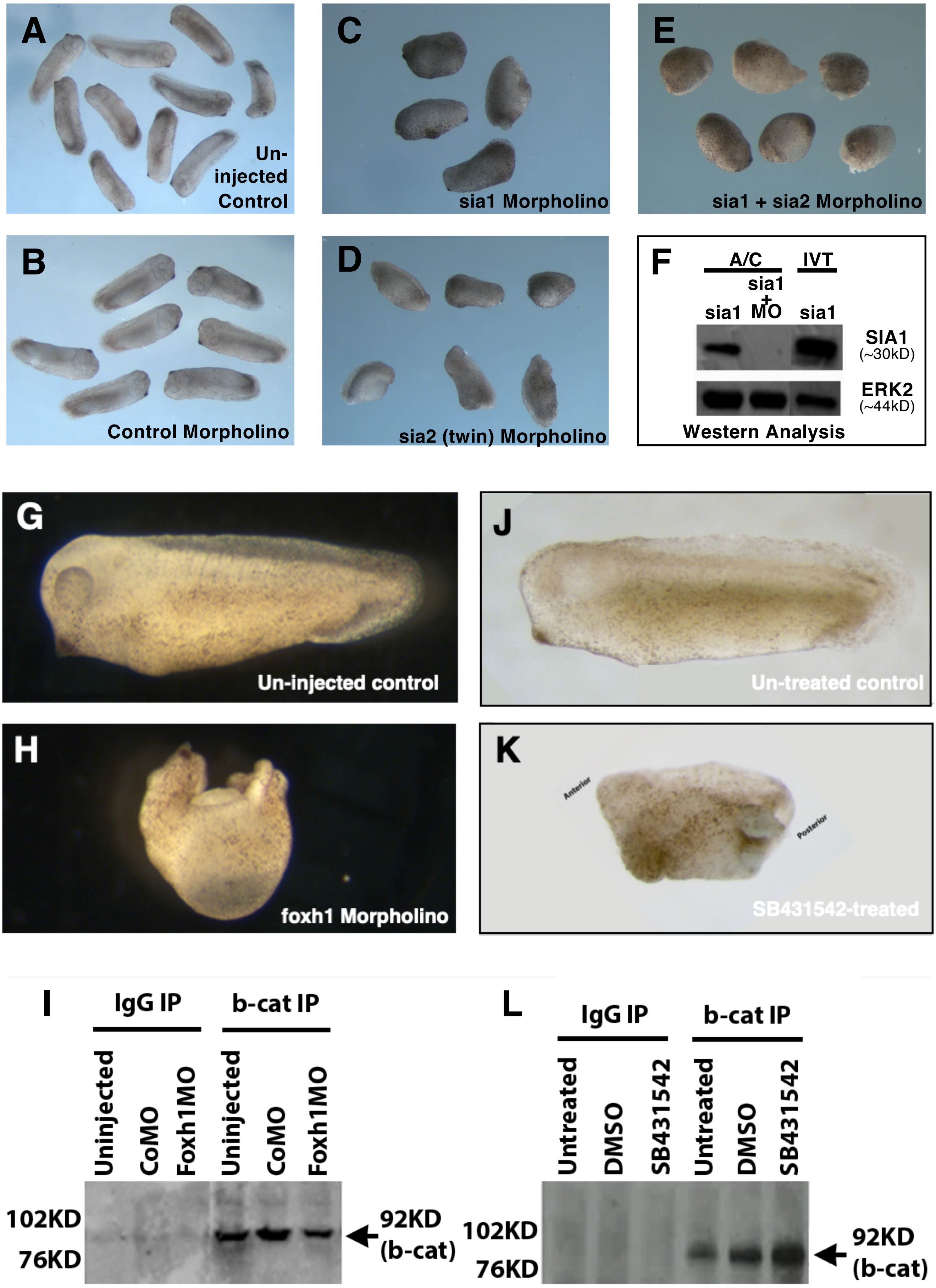
A
Un-
injected
Control
C
sia1 Morpholino
E
sia1 + sia2 Morpholino
B
Control Morpholino
D
sia2 (twin) Morpholino
F
A/C
IVT
sia1
sia1 + MO
sia1
SIA1
(~30kD)
ERK2
(~44kD)
Western Analysis
G
Un-injected control
J
Un-treated control
H
foxh1 Morpholino
K
Anterior
Posterior
SB431542-treated
I
IgG IP
b-cat IP
Uninjected
CoMO
Foxh1MO
Uninjected
CoMO
Foxh1MO
102KD
76KD
92KD
(b-cat)
L
IgG IP
b-cat IP
Untreated
DMSO
SB431542
Untreated
DMSO
SB431542
102KD
76KD
92KD
(b-cat)

Suppl. Fig. 4

Suppl. Figure 4: Specificity of siamois Morpholino, FoxH1 Morpholino and SB431542 TGFβ inhibitor, Related to Figures 3 and 4

A-E) *Xenopus tropicalis* embryos were injected into the two-dorsal blastomeres of the four-cell embryo with 10ng of either control Morpholino MO (B; N=45), sia1 MO (C; N=27) or sia2 (twn) MO (D; N=32); or both sia1 and sia2 MO (F; N=22)(A is un-injected control, see Transparent Methods for MO sequences). Note lack of A-P and D-V patterning in the single-injected morphants (C, D) and more severe effects in the double-injected morphants (E). (F) *Xenopus laevis* embryos were injected at one-cell stage into the animal pole with 10pg of mRNA encoding *Xenopus tropicalis* sia1 (Haramoto et al., 2017) or combined with *Xenopus tropicalis* sia1 MO, as indicated. Animal caps explants (A/C) were excised at stage 8 and explants cultured until stage 12 to monitor protein expression using an anti-rabbit Sia antibody (Sudou et al., 2012). Note that MO efficiently blocks Sia protein production. IVT: In Vitro Translation. (G-I) *Xenopus tropicalis* Foxh1 MO (Chiu et al., 2014) was injected into *Xenopus tropicalis* embryos (see Transparent Methods) and embryos collected when control un-injected embryos (G) reached stage 32. (H) Foxh1 morphant display severe A-P and D-V defects. (I) ChIP qPCR analysis; i.e. immunoprecipitation for chromatin-associated β-catenin protein (see Transparent Methods). (J-L) Embryos were treated with TGFβ inhibitor SB431542 (see Transparent Methods) until stage 32 when control untreated embryos reached stage 32 (J). (K) SB431542-treated embryos lack distinctive A-P patterning and (L) ChIP analysis performed using conditions and reagents as in (I).

TRANSPARENT METHODS

Embryo manipulations:

Xenopus tropicalis embryos were obtained by *in vitro* fertilization (del Viso and Khokha, 2012) and staged according to Nieuwkoop and Faber (1967). The fertilized embryos were either injected with morpholinos (MOs) and/or mRNA or treated with chemical inhibitors (see below), as indicated. The injected embryos were cultured in 0.1x Marc's Modified Ringer (MMR) at 28°C. Sequences of MOs obtained from Gene Tools (Philomath, Oregon, United States) were as follow:

- CoMO: 5'-CCTCTTACCTCAGTTACAATTTATA-3' (Khokha et al., 2002);
- *Xenopus tropicalis* (Xt) β-catenin (ctnnb1) MO (Khokha et al., 2002): 5'-TTTCAACAGTTTCCAAAGAACCAGG-3';
- Xt foxh1 MO (Chiu et al., 2014): 5'-TCATCCTGAGGCTCCGCCCTCTCTA-3';
- Xt sia1-1 MO: 5' GCTCCATTTCAGCCTCACAGGTCAT 3' (X. tropicalis equivalent to X.laevis sia1 MO from Bae et al., 2011, shown in Fig. 3 and Suppl. Fig. 4);
- Xt sia1-2 MO: 5' TTCGCCTCACAGGTCATGTCTGTC 3' (X. tropicalis equivalent to X. laevis sia1 MO from Ishibashi et al., 2008, used as additional control, not shown);
- Xt sia2-1 MO: 5' GCTCAAGCTCAGAGTCACAAGTCAT 3' (X. tropicalis equivalent of X. laevis twn MO from Bae et al., 2011, used as additional control, not shown);
- Xt sia2-2 MO: 5' CTCAGAGTCACAAGTCATCCTTGAA 3' (X. tropicalis equivalent of X. laevis twn MO from Ishibashi et al., 2008, shown in Fig.3 and Suppl. Fig. 4).

The two sia1 and the two sia2 MOs were tested in pilot experiments and confirmed to induce the expected phenotype (Bae et al., 2011). Since they induced these phenotypes at lower injection amounts or in a higher percentage of embryos when injected at the same amount (2.5ng per blastomere, 5ng per embryo), we continued our experiments with sia1-1 and sia2-2 MOs. Capped mRNA was synthesized using mMESSAGE mMACHINE Kit (Ambion) according

to manufacturer instructions. β-catenin plasmid (Yost et al., 1996) and constitutively active siamois VP16-sia plasmid (Kessler, 1997) were linearized with NotI and *in vitro* transcribed with SP6. The VP16-siamois fusion construct was used as an additional rescue control for the sia1 and sia2 Morpholino knockdown (e.g. Fig.3B), since its sequence was not targeted by any of the four MO used to knockdown *sia1* and *sia2* gene expression. The Nodal/TGFβ-signaling inhibitor SB431542 (Tocris Bioscience) was reconstituted to 10mM and diluted to 100μM in the culture medium. Four-cell stage embryos were immersed in 100μM SB431542 in 1/9xMMR and cultured at 25°C until mock (solvent)-treated siblings reached desired stage (see also Chiu et al., 2014).

Relevant regulatory standards

Experiments conducted at the University of Aberdeen were initially assessed and approved and in 2017 reviewed and renewed each time first by the University of Aberdeen Ethical Review Committee and then by the United Kingdom Home Office Inspector. All animal experiments were subsequently carried out under license from the United Kingdom Home Office: PPL 60-04376 (until 19 September 2017) and PPL PA66BEC8D (since 20 September 2017). For experiments conducted at the University of California Irvine, animals were raised and maintained in accordance with the University of California, Irvine Institutional Animal Care Use Committee (IACUC) and guided by husbandry methods developed by the National Xenopus Resource (Marine Biological Laboratory, Woods Hole, MA).

RNA extraction and RNA expression analysis with qPCR and RNA-seq

Total RNA was isolated from whole embryos using the RNeasy Mini Kit, according to manufacturer's instructions (QIAGEN) for processing of animal tissues (see also Lee-Liu et al., 2012; Nakamura et al., 2016). The abundance of RNAs was determined using a LightCycler 480 and SYBR Green I Master Reagents (Roche). Relative expression levels of genes was determined using ΔΔC(t) or Livak method (Taneyhill and Adams, 2008). For the RNA-seq analysis

of the functional β-catenin experiments Illumina TruSeq RNA libraries were constructed and sequenced using Illumina HiSeq 2000 at the Earlham Institute, Norwich, UK. For the RNA-seq analysis of the foxh1 MO knockdown and the Nodal/TGFβ signaling inhibition experiments total RNA was extracted from ~25-30 control and experimental early gastrula embryos using the acid guanidinium thiocyanate phenol chloroform method (Chomczynski and Sacchi, 1987) followed by selective precipitation of RNA using 2.5M LiCl. The quality of the RNA was examined using an Agilent BioAnalyzer 2100 instrument. 1µg of total RNA was subjected to oligo(dT) selection to extract polyadenylated RNA, which was then chemically fragmented and libraries were generated for single-end sequencing according to Illumina's RNA-seq sample preparation kit (see also Chiu et al., 2014).

β-catenin ChIP and β-catenin ChIP-seq

β-catenin ChIP qPCR and ChIP-seq experiments were conducted using anti-β-Catenin (Ctnnb1) antibody (H-102). *Xenopus tropicalis* embryos were harvested at stage 7 and fixed at room temperature with 1% formaldehyde in phosphate-buffered saline (PBS) for 45 minutes. Immediately after fixation, the embryos were incubated with 125 mM glycine/PBS for 10 minutes and washed three times with ice-cold PBS for 5 minutes. Batches of 50 embryos were snap-frozen in liquid nitrogen and stored at -80°C for future use. For the following procedures, all solutions and samples were kept on ice. RIPA buffer (50 mM Tris pH 7.4, 150 mM NaCl, 1 mM EDTA, 1% IGEPAL CA-630, 0.25% Sodium deoxycholate, 0.1% SDS, 0.5 mM DTT) supplemented with Protease Inhibitor Cocktail (Sigma, P8340) was added to frozen embryos. Embryos were thawed on ice for 10-15 minutes, homogenized, and then kept on ice for 10 minutes. After re-homogenization, the embryo extracts were transferred to TPX microtubes (Diagenode) and sonicated during 25 cycles with 30 seconds ON/30 seconds OFF at high power setting using the Bioruptor Plus Instrument (Diagenode). The sonicated samples were centrifuged at 14,000 rpm for 10 minutes at 4°C, and the supernatant was transferred to a 1.5ml tube for subsequent use for ChIP and input samples. A small aliquot of the supernatant

was used for checking chromatin shearing. The input samples were stored at -20°C for later usage. The supernatant for ChIP were incubated for 1 hour at 4°C with Dynabeads Protein G (Life technologies) that had been blocked with 5% BSA/PBS for 1 hour at 4°C. After snap-spin, the supernatant was transferred to a 1.5ml safe-lock tube and incubated with antibodies (2 ug) overnight at 4°C. On the following day, chromatin was precipitated with 5% BSA/PBS-blocked Dynabeads Protein G for 1 hour at 4°C and then the beads were successively washed with ChIP buffer 1 (20 mM Tris pH 8.0, 150 mM NaCl, 2 mM EDTA, 1% Triton X-100, 0.1% SDS), ChIP buffer 2 (20 mM Tris pH 8.0, 500 mM NaCl, 2 mM EDTA, 1% Triton X-100, 0.1% SDS), ChIP buffer 3 (10 mM Tris pH 8.0, 250 mM LiCl, 1 mM EDTA, 1% IGEPAL CA-630, 1% Sodium deoxycholate), ChIP buffer 4 (10 mM Tris pH 8.0, 1 mM EDTA) for 5 minutes each. Chromatin was eluted from the beads with elution buffer (50 mM Tris pH 8.0, 10 mM EDTA, 1% SDS) for 20 minutes in a Thermoshaker (65°C, 900 rpm). At this stage, the frozen input samples were supplemented with elution buffer. ChIP and input samples were incubated with RNase A at 37°C for 30 minutes. The samples were then added with NaCl and incubated for over 16 hours in a Thermoshaker (65°C, 900 rpm). The samples were further treated with proteinase K for 2 hours in a Thermoshaker (65°C, 900 rpm). The de-crosslinked DNA fragments were purified with phenol:chloroform:isoamylalcohol and precipitated in ethanol using 50 embryos for qPCR (e.g. Fig.4J,K). For sequencing, sheared chromatin was collected from approximately 25,000 stage 7 embryos. Each ChIP DNA and input control DNA was purified using MinElute Reaction Cleanup Kit (QIAGEN) and pooled to one sample. The purified DNA was quantified using Qubit dsDNA HS Assay Kits (Life technologies) by Qubit 2.0 Fluorometer (Life technologies). Illumina TrueSeq ChIP libraries were constructed from the ChIP DNA and the input control DNA samples and sequenced using 50 bp single-end reads by Illumina HiSeq 2500 at the Earlham Institute, Norwich, UK (see also Akkers et al., 2012; Nakamura et al., 2016).

Foxh1 ChIP-seq experiment

Foxh1 ChIP-seq experiments were carried out with a custom anti-Foxh1 antibody (Chiu et al., 2014) using 4 µg of antibody per 100-embryo-equivalents of chromatin for ChIP. Embryos were cultured in 1/9X MMR at 25°C until the indicated stage and fixed in 1% formaldehyde at room temperature for 45 minutes with gentle rocking. Crosslinking reactions were neutralized by the removal of the formaldehyde solution and incubation with 1ml 0.125M glycine solution for 10 minutes on ice. Embryos were then washed with cold RIPA buffer (50 mM Tris-HCl pH7.4, 150mM NaCl, 1mM EDTA, 0.25% sodium deoxycholate, 1%NP40, 0.1% SDS, 0.5mMDTT, and Roche cOmplete protease inhibitor cocktail), flash frozen, and stored at -80°C. The fixed embryos were homogenized in RIPA buffer and incubated on ice for 10 minutes. Samples were then microfuged at 14,000 rpm for 15 minutes at 4°C. Pellets were resuspended in RIPA buffer and sonicated on ice using a Branson Digital Sonifier 450 resulting in an average fragment size between 200-500bp. The samples were microfuged at 14,000 rpm for 20 minutes at 4°C to remove insoluble cellular debris. The chromatin was then "pre-cleared" by incubating with Protein A-coated Dynabeads (Invitrogen) for 2 hour at 4°C with rotation. Antibodies were pre-bound to blocked Protein A Dynabeads by incubating at 4°C for 30 min. A sample of sheared chromatin was frozen for use as an input control. Pre-cleared chromatin was added to antibody-bound Dynabeads, and incubated overnight at 4°C on an end-over-end rotator. The next day, the beads were washed for 20 minutes each with ice-cold ChIP wash solution I (50mM HEPES-KOH pH7.5, 2mM EDTA, 150mM NaCl, 0.1% sodium deoxycholate, 1% Triton X-100, 1mM DTT, and 0.4mM PMSF), ChIP wash solution II (50mM HEPES-KOH pH7.5, 2mM EDTA, 500mM NaCl, 0.1% sodium deoxycholate, 1% Triton X-100, 1mM DTT, and 0.4mM PMSF), ChIP wash solution III (0.25 M LiCl, 1 mM EDTA, 10 mM Tris-HCl pH 8.0, 0.5% NP-40, 0.5% sodium deoxycholate, 1 mM DTT, and 0.4 mM PMSF), and TE (10mM Tris, 1mM EDTA, 1 mM DTT, and 0.4 mM PMSF). The DNA was then eluted with TE buffer containing 1% SDS, and reverse-crosslinked at 65°C overnight. The sonicated input control was diluted 3-fold with elution buffer, and also incubated at 65°C. All samples were treated with

RNAse A, Proteinase K, phenol/chloroform extracted, and ethanol precipitated overnight. DNA pellets were resuspended in Qiagen EB solution. 10-30ng of total ChIP DNA was used for library construction using the NEXTflex ChIP-seq kit (Bioo Scientific). Sequencing was performed using the Illumina HiSeq 2500 and 50bp single-end reads were obtained (see also Charney et al., 2017; Chiu et al., 2014).

Bioinformatics:

RNA-seq differential expression analysis

Maternal Wnt/β-catenin-regulated transcriptome RNA sequencing data was quality control checked using FastQC (Andrews, 2015, version 0.11.3), aligned to the *Xenopus tropicalis* V9 reference genome (James-Zorn et al., 2015; Karpinka et al., 2015) using STAR (Dobin et al., 2013, version 2.4.0), converted to bam format and sorted using SAMtools (Li et al., 2009, version 1.2), and quantified at gene regions using HTSeq (Anders and Huber, 2010, version 0.6.1).

RNA sequencing data from Foxh1 MO and TGFβ inhibitor (SB431542) experiments were quality control checked using FastQC (Andrews, 2015, version 0.11.3) and TrimGalore! (Krueger, 2015, version 0.4.0), aligned to the *Xenopus tropicalis* V9 reference genome (James-Zorn et al., 2015; Karpinka et al., 2015) using HISAT2 (Kim et al., 2015, version 2.1.0), converted to bam format and sorted using SAMtools (Li et al., 2009, version 1.2), and quantified at gene regions using featureCounts (Liao et al., 2014, from the Subread package version 5.0-p1) with the parameter enabled to split multi-mapped reads as a fraction across hits.

In all cases, differential expression analysis was carried out with DESeq2 (Love et al., 2014, version 1.14.1) using generalized linear models with the LRT function. For Foxh1 and SB data sets, significance was identified by an FDR < 0.1. For the β-catenin samples, two models were used, incorporating the data from the rescue strain, genes with an FDR < 0.1 were selected, and an intersect of these genes between the two models was used to identify significance.

ChIP Sequencing analysis

ChIP sequencing peak data for zygotic β-catenin (st.10) was obtained from Nakamura et al. (2016). Previously published raw ChIP sequencing data for Foxh1 from Chiu et al. (2014) and Charney et al. (2017) was downloaded from the Gene Expression Omnibus using SRA toolkit (Alnasir and Shanahan, 2015, www.ncbi.nlm.nih.gov/sra, version 2.8.2). To ensure consistency in the analysis, previously published and new Foxh1 sequencing data (stage 7) was analyzed in an identical manner to Nakamura et al. (2016). In short, this encompassed quality filtering with FastQC (Andrews, 2015, version 0.11.3) and TrimGalore! (Krueger, 2015, version 0.4.0), alignment to the *Xenopus tropicalis* v9 reference genome (James-Zorn et al., 2015; Karpinka et al., 2015) using BWA aln (Li, 2013, version 0.7.12), conversion to sam format with BWA samse (Li, 2013, version 0.7.12), conversion to bam format with SAMtools (Li et al., 2009, version 1.2) incorporating removal of unmapped reads and non-primary alignments, peak calling independently with MASC2 (Zhang et al., 2008, version 2.1.20160309) and SPP (Kharchenko et al., 2008, version 1.14), before consensus peak calling with IDR (Li et al., 2011, version 2.0.2), and finally identifying common peaks between replicates, and between Foxh1 and β-catenin ChIP samples, using BEDTools (Quinlan, 2014; Quinlan and Hall, 2010, version 2.26.0). Peak selection was based on any genomic region that had overlapping regions of any length between two peak files. Closest adjacent genes were identified using BEDTools (Quinlan, 2014; Quinlan and Hall, 2010, version 2.26.0). Heatmaps of peak regions were created using Homer (Heinz et al., 2010, version 4.8.3). De-novo motif analysis was performed using Homer (Heinz et al., 2010, version 4.8.3) and MEME-ChIP (Machanick and Bailey, 2011, version 4.11.2).

SUPPLEMENTAL REFERENCES

Akkers, R.C., Jacobi, U.G., and Veenstra, G.J.C. (2012). Chromatin immunoprecipitation analysis of Xenopus embryos. Methods Mol Biol *917*, 279-292.

Alnasir, J., and Shanahan, H.P. (2015). Investigation into the annotation of protocol sequencing steps in the sequence read archive. Gigascience *4*, 23.

Anders, S., and Huber, W. (2010). Differential expression analysis for sequence count data. Genome Biology *11*, R106.

Andrews, S. (2015).

Bae, S., Reid, C.D., and Kessler, D.S. (2011). Siamois and Twin are redundant and essential in formation of the Spemann organizer. Dev Biol *352*, 367-381.

Charney, R.M., Forouzmand, E., Cho, J.S., Cheung, J., Paraiso, K.D., Yasuoka, Y., Takahashi, S., Taira, M., Blitz, I.L., Xie, X., *et al.* (2017). Foxh1 Occupies cis-Regulatory Modules Prior to Dynamic Transcription Factor Interactions Controlling the Mesendoderm Gene Program. Dev Cell *40*, 595-607 e594.

Chiu, W.T., Charney Le, R., Blitz, I.L., Fish, M.B., Li, Y., Biesinger, J., Xie, X., and Cho, K.W. (2014). Genome-wide view of TGFbeta/Foxh1 regulation of the early mesendoderm program. Development *141*, 4537-4547.

Chomczynski, P., and Sacchi, N. (1987). Single-step method of RNA isolation by acid guanidinium thiocyanate-phenol-chloroform extraction. Anal Biochem *162*, 156-159.

del Viso, F., and Khokha, M. (2012). Generating diploid embryos from Xenopus tropicalis. Methods Mol Biol *917*, 33-41.

Dobin, A., Davis, C.A., Schlesinger, F., Drenkow, J., Zaleski, C., Jha, S., Batut, P., Chaisson, M., and Gingeras, T.R. (2013). STAR: ultrafast universal RNA-seq aligner. Bioinformatics *29*, 15-21.

Haramoto, Y., Saijyo, T., Tanaka, T., Furuno, N., Suzuki, A., Ito, Y., Kondo, M., Taira, M., and Takahashi, S. (2017). Identification and comparative analyses of Siamois cluster genes in Xenopus laevis and tropicalis. Dev Biol *426*, 374-383.

Heinz, S., Benner, C., Spann, N., Bertolino, E., Lin, Y.C., Laslo, P., Cheng, J.X., Murre, C., Singh, H., and Glass, C.K. (2010). Simple combinations of lineage-determining transcription factors prime cis-regulatory elements required for macrophage and B cell identities. Mol Cell *38*, 576-589.

Ishibashi, H., Matsumura, N., Hanafusa, H., Matsumoto, K., De Robertis, E.M., and Kuroda, H. (2008). Expression of Siamois and Twin in the blastula Chordin/Noggin signaling center is required for brain formation in Xenopus laevis embryos. Mech Dev *125*, 58-66.

James-Zorn, C., Ponferrada, V.G., Burns, K.A., Fortriede, J.D., Lotay, V.S., Liu, Y., Brad Karpinka, J., Karimi, K., Zorn, A.M., and Vize, P.D. (2015). Xenbase: Core features, data acquisition, and data processing. Genesis *53*, 486-497.

Karpinka, J.B., Fortriede, J.D., Burns, K.A., James-Zorn, C., Ponferrada, V.G., Lee, J., Karimi, K., Zorn, A.M., and Vize, P.D. (2015). Xenbase, the Xenopus model organism database; new virtualized system, data types and genomes. Nucleic Acids Res *43*, D756-763.

Kessler, D.S. (1997). Siamois is required for formation of Spemann's organizer. Proc Natl Acad Sci U S A *94*, 13017-13022.
Kharchenko, P.V., Tolstorukov, M.Y., and Park, P.J. (2008). Design and analysis of ChIP-seq experiments for DNA-binding proteins. Nat Biotechnol *26*, 1351-1359.
Khokha, M.K., Chung, C., Bustamante, E.L., Gaw, L.W., Trott, K.A., Yeh, J., Lim, N., Lin, J.C., Taverner, N., Amaya, E.*, et al.* (2002). Techniques and probes for the study of Xenopus tropicalis development. Dev Dyn *225*, 499-510.
Kim, D., Langmead, B., and Salzberg, S.L. (2015). HISAT: a fast spliced aligner with low memory requirements. Nat Methods *12*, 357-360.
Krueger, F. (2015).
Lee-Liu, D., Almonacid, L.I., Faunes, F., Melo, F., and Larrain, J. (2012). Transcriptomics using next generation sequencing technologies. Methods Mol Biol *917*, 293-317.
Li, H. (2013). Aligning sequence reads, clone sequences and assembly contigs with BWA-MEM. arXiv, p.1303.3997v2. . arXiv, 1303.3997v1302.
Li, H., Handsaker, B., Wysoker, A., Fennell, T., Ruan, J., Homer, N., Marth, G., Abecasis, G., Durbin, R., and Genome Project Data Processing, S. (2009). The Sequence Alignment/Map format and SAMtools. Bioinformatics *25*, 2078-2079.
Li, Q., Brown, J.B., Huang, H., and Bickel, P.J. (2011). Measuring reproducibility of high-throughput experiments. The Annals of Applied Statistics *5*, 1752-1779.
Liao, Y., Smyth, G.K., and Shi, W. (2014). featureCounts: an efficient general purpose program for assigning sequence reads to genomic features. Bioinformatics *30*, 923-930.
Love, M.I., Huber, W., and Anders, S. (2014). Moderated estimation of fold change and dispersion for RNA-seq data with DESeq2. Genome biology *15*, 550.
Machanick, P., and Bailey, T.L. (2011). MEME-ChIP: motif analysis of large DNA datasets. Bioinformatics *27*, 1696-1697.
Nakamura, Y., de Paiva Alves, E., Veenstra, G.J., and Hoppler, S. (2016). Tissue- and stage-specific Wnt target gene expression is controlled subsequent to beta-catenin recruitment to cis-regulatory modules. Development *143*, 1914-1925.
Nieuwkoop, P.D., and Faber, J. (1967). Normal Table of Xenopus laevis (Daudin): a systematical and chronological survey of the development from the fertilized egg to the end of metamorphosis. (New York and London: Garland Publishing, Inc.).
Owens, N.D.L., Blitz, I.L., Lane, M.A., Patrushev, I., Overton, J.D., Gilchrist, M.J., Cho, K.W.Y., and Khokha, M.K. (2016). Measuring Absolute RNA Copy Numbers at High Temporal Resolution Reveals Transcriptome Kinetics in Development. Cell Rep *14*, 632-647.
Quinlan, A.R. (2014). BEDTools: The Swiss-Army Tool for Genome Feature Analysis. Curr Protoc Bioinformatics *47*, 11 12 11-34.
Quinlan, A.R., and Hall, I.M. (2010). BEDTools: a flexible suite of utilities for comparing genomic features. Bioinformatics *26*, 841-842.

Sudou, N., Yamamoto, S., Ogino, H., and Taira, M. (2012). Dynamic in vivo binding of transcription factors to cis-regulatory modules of cer and gsc in the stepwise formation of the Spemann-Mangold organizer. Development *139*, 1651-1661.

Taneyhill, L.A., and Adams, M.S. (2008). Investigating regulatory factors and their DNA binding affinities through real time quantitative PCR (RT-QPCR) and chromatin immunoprecipitation (ChIP) assays. Methods Cell Biol *87*, 367-389.

Yost, C., Torres, M., Miller, J.R., Huang, E., Kimelman, D., and Moon, R.T. (1996). The axis-inducing activity, stability, and subcellular distribution of beta-catenin is regulated in Xenopus embryos by glycogen synthase kinase 3. Genes Dev *10*, 1443-1454.

Zhang, Y., Liu, T., Meyer, C.A., Eeckhoute, J., Johnson, D.S., Bernstein, B.E., Nusbaum, C., Myers, R.M., Brown, M., Li, W., *et al.* (2008). Model-based analysis of ChIP-Seq (MACS). Genome biology *9*, R137.
